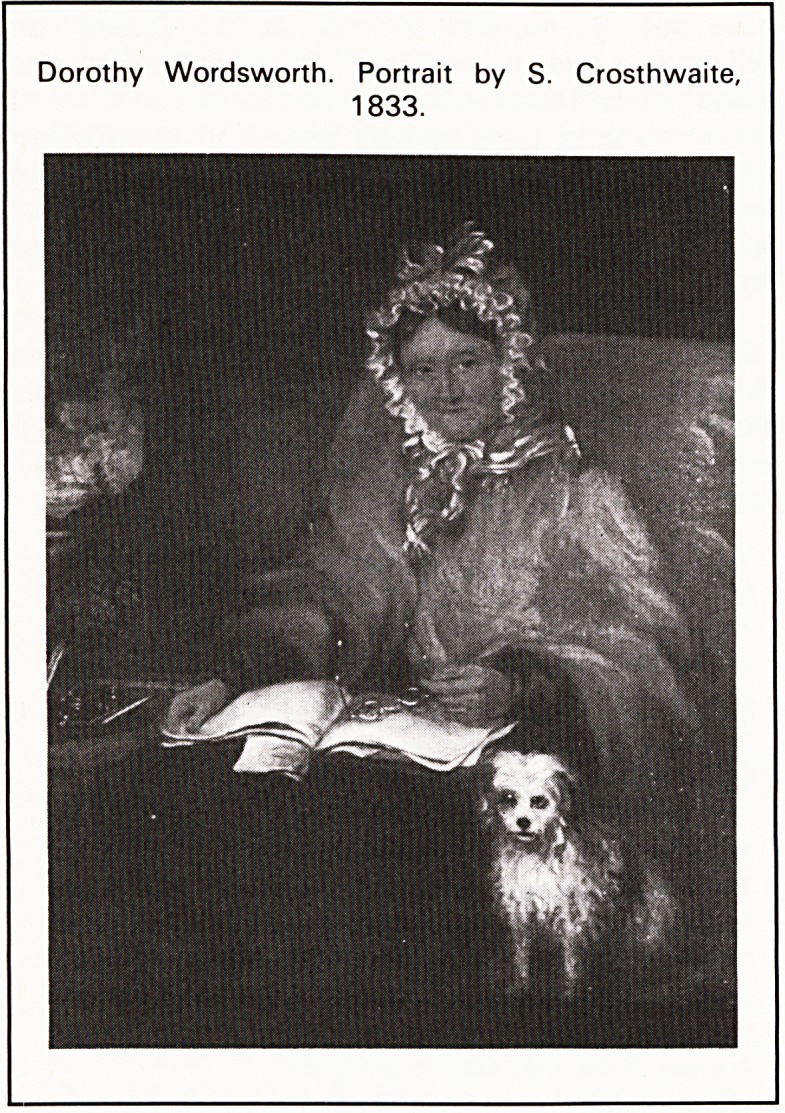# Wordsworth, Presidential Address

**Published:** 1985-01

**Authors:** Ian Bailey


### Wordsworth

### Presidential Address to the Bristol Medico-Chirurgical Society, 10th October 1984

**Ian Bailey, M.D., F.R.C.P.**

I will tell you the story tonight of a man born in Cockermouth, Cumberland, in 1770, orphaned in childhood, educated at Hawkshead Grammar School and St. John's College, Cambridge, who travelled widely in Britain and Europe, became an admirer of the new revolutionary regime in France, fathered an illegitimate daughter in Orléans, who made poetry his vocation, who came to Bristol and later returned to the Lake District and who, from the first flush of revolutionary fervour came to a dignified old age in Rydal, Westmorland as Queen Victoria's Poet Laureate, a Tory supporter, opponent of reform and adherent to the established church.

William Wordsworth. Portrait by Henry Inman, 1884.

There are several reasons for choosing this subject. I have heard some twenty Presidential Addresses to this Society, from Mr. Robert Cooke's 'Ship-Shape and Bristol Fashion' to Dr. Norman Tricks' 'Man and Medicine on Mendip'. Sometimes the President has chosen a topic of local or general medical history, at other times a person: St. Peter, Louis Napoleon, Jenner and Beethoven. My subject was born in the same year as Beethoven, and wrote 'The Child is Father of the Man' – the words with which Dr. Beryl Corner began her address on Beethoven, then used to emphasise the importance of childhood, tonight used to suggest a significance for old age.

To understand how he came to Bristol, we need to go back to the beginning. Wordsworth was born on 7th April, 1770, the second child of John Wordsworth, lawyer and agent for Sir James Lowther in West Cumberland. He was attracted to the River Derwent flowing beneath the terrace at the bottom of the garden:

> '. . . the fairest of rivers, loved
> To blend his murmurs with my nurse's song,
> And from his alder shades and rocky falls,
> And from his fords and shallows, sent a voice
> That flowed along my dreams'.

The river had given him:

> 'a foretaste, a dim earnest of the calm
> That nature breathes among the hills and groves.'

He enjoyed his childhood, and later wrote:

> 'Fair seed time had my soul, and I grew up
> Fostered alike by beauty and by fear:
> Much favoured in my birthplace.'

He was devoted to his mother who died from pneumonia just before his eighth birthday, and wrote:

> 'For him in one dear Presence, there exists
> A nature which irradiates and exalts'

and Wordsworth came to transfer to Nature the affection and love which he had felt for his mother.

His younger sister, Dorothy, was his playmate in early childhood. Soon after her mother's death she was sent to relatives in Halifax, Yorkshire, and was not to see William for another 9 years. In 1779, aged nine, William and his 11-year-old brother attended the Grammar School in Hawkshead and lodged with Ann Tyson. Hawkshead was a prosperous woollen town whose Grammar School was founded by Edwin Sandys, Archbishop of York in 1585, and the present building dates from 1675. There were about a hundred boys in the school and it had at that time a high reputation and many pupils entered Cambridge. School hours were from 6 a.m. to 11 a.m. and 1 p.m. to 5 p.m. William found time to wander about the countryside, to visit Furness Abbey and to sit on the benches talking to the old people – for there were old people in the eighteenth century. In Hawkshead in one year of Wordsworth's schooldays half the registered burials were of people aged 80 to 89.

William's father died in the Christmas holidays of 1783 and the children came under the guardianship of their uncles in Whitehaven and Penrith. There was a reunion with Dorothy in the summer of 1787, and in October Wordsworth set off by coach for Cambridge. He had a 'nook obscure' in the first court of St. John's College above the kitchens, and the window of his study looked out on to Trinity College Chapel:

'And from my pillow, looking forth
By light of moon or favourite stars, I could behold
The antechapel where the statue stood
Of Newton with his prism and his silent face,
The marble index of a mind forever
Voyaging through strange seas of thought, alone.'

He joined a party in the rooms formerly occupied by his greatest hero Milton, and became intoxicated for the first and probably the last time in his life—

'And gratitude grew dizzy in a brain
Never excited by the fumes of wine
Before that hour, or since.'

But he was still able to run 'ostrich like' to reach chapel by 9 p.m.

Wordsworth was placed 1st Class at the end of his first term, 2nd Class at the end of his first year. He describes the fascination of a return to the Lake District for the summer vacation:

'With exultation at my feet I saw
Lake, islands, promontories, gleaming bays,
A universe of Nature's fairest forms.'

And later, as he left a party at a farmhouse near Hawkshead at 4 a.m. and saw the sun rising over Helvellyn:

'The morning rose, in memorable pomp,
Glorious as e'er I had beheld. In front
The sea lay laughing at a distance; near
The solid mountains shone, bright as the clouds,
Grain-tinctured, drenched in empyrean light;
And in the meadows and in the lower grounds
Was all the sweetness of a common dawn.'

Wordsworth seemed thereafter to lose interest in Cambridge—

'A feeling that I was not for that hour, nor for that place'

and created his own pattern of study. He became anti-clerical and anti-establishment. The Master of St. John's College died in March 1789. It was the custom for literary minded students to pin on the coffin words of appreciation. Wordsworth refused to do this. In his last summer vacation just before finals he set off on a three month walking tour through France to the Alps, failed to sit the full examination and received an unclassified B.A. in January 1791. Describing his last two years and his sense of detachment, he says that he decided to live 'like a lodger in that house of letters'.

The trip to France was with a fellow student, Robert Jones, in July 1790, almost a year after the storming of the Bastille and the start of the French Revolution. The whole nation was mad with joy and they joined in the festivities in each village. They walked 20 miles a day, reached and were disappointed by Switzerland and travelled by boat down the Rhine:

'Bliss was it in that dawn to be alive
But to be young was very heaven'.

Wordsworth had no idea what to do after his degree. Neither the Church, the Law or the Army had any appeal. He drifted round London and in the summer of 1791 spent four months walking in North Wales with Jones. He wondered whether to lead the life of a tramp! His guardians, despairing, pressed him to take a curacy, and for a short while he did attend lectures in Hebrew and Oriental languages as a preparation for clerical life. But in December he travelled again to France, this time by coach through Paris to Orléans. He met Annette Vallon whose father had been a surgeon in Blois, and spent some time with her in Orléans and Blois. Wordsworth left Orléans in October 1792 possibly because it was then dangerous to remain in France. The King had been deposed following the storming of the Tuilleries that August. The Commune, led by Danton, Marat and Robespierre had encouraged the September Massacre. Wordsworth reached England in December 1792 and his daughter Caroline was born on 15th December 1792. The Annette affair was never publicly known during Wordsworth's lifetime, was not referred to directly in the poem of his life, The Prelude, was not mentioned in his official biography, and only came to light in the 1920s when letters from Annette were discovered, undelivered and unread, in departmental archives in the Loire.

Wordsworth spent the next six months in London, published his first poems in 'Descriptive Sketches' and 'An Evening Walk' in January 1793. Dorothy wrote that they contained many faults, the chief being obscurity, and they received little attention and later savage reviews.

On 8th January 1793 Louis XVI and Marie Antoinette were executed. England declared war on France. Wordsworth was unable to return to France until a brief interlude in the war following the Peace of Amiens in 1802.

He became involved in radical politics and supported the idea of an English Revolution, but more in spirit than deed. In an unpublished letter to the Bishop of Llandaff, he attacked the British Monarchy. He had no money, no home, no training, had been disowned by his relatives, except the adoring sister Dorothy whom he was forbidden to visit at the Parsonage in Norwich where she was caring for her uncle's family. Both William and Dorothy longed for a cottage which they could share.

Wordsworth was invited to join William Calvert, a wealthy school friend, on a tour of the West Country. The coach was involved in an accident on Salisbury Plain. Calvert travelled north on horseback and Wordsworth on foot. He had a visionary experience on Salisbury Plain, visited Tintern, stayed with Jones, now a cleric in North Wales, and reached the Calvert home, Windy Brow, Keswick, at the end of 1793. In February 1794 he met Dorothy at Halifax. After six weeks with relatives they eloped on foot heading for the Lake District. Dorothy wrote: 'I walked with my brother at my side from Kendal to Grasmere, 18 miles, and afterwards from Grasmere to Keswick 15 miles through the most delightful country that ever was seen.' They spent the early summer of 1794 at Windy Brow, and visited Cockermouth, their old home now empty and the terrace walk buried and choked up with the old privet hedge. Dorothy returned to relatives in the summer and William stayed at Windy Brow as a companion to William Calvert's younger brother Raisley, who at 21 was suffering from tuberculosis from which he died in January 1795 leaving Wordsworth £900.

Grasmere, Cumbria.

Wordsworth returned to London and shared rooms with Basil Montagu, a Cambridge contemporary who was studying for the bar and taking pupils to pay his way and to help support his motherless 2-year-old son. Two pupils, John Frederick and Azariah Pinney, came from Bristol and offered Wordsworth their family's country home, Racedown, rent free. So Dorothy and William moved, with Montagu's son, there in September 1795. At last there was some stability after years of turmoil and uncertainty.

What, meantime, had been happening in Bristol? Coleridge, Southey and other young radicals had decided to get away from a reactionary England to found a Utopian community on the banks of the Susquehanna. Twelve young men and twelve young women would set up an agricultural community. The scheme was called Pantisocracy.

Coleridge was born in Ottery St. Mary in 1772, the tenth child of the vicar. He was educated at Christ's Hospital where he formed a lifelong friendship with Charles Lamb, and at Jesus College, Cambridge. Rejected by a girl friend, he enlisted in the 1st Regiment of the Light Dragoons under the name of Silas Tomkyn Comberback. He wrote love letters for his fellows and surprised his officers by his knowledge of Greek. After several months he was persuaded to return to Cambridge but left in 1794 without a degree. Spending three weeks in Oxford he met Southey at Balliol and there was conceived the idea of Pantisocracy.

Southey, a Bristolian, was born in Wine Street in 1774 and brought up from the age of two by an eccentric aunt who insisted that he slept in her bed and though waking at 6 a.m. was not allowed to rise until she did at 11 a.m. He was educated at Westminster and was expelled after making an attack on the school and corporal punishment in the magazine 'The Flagellant' which he had founded. Balliol accepted him. He was involved in radical politics and one of his first acts of protest was to go to formal dinner with his hair unpowdered, for Pitt, hated by radicals, had just put a tax on powder. He found a kindred spirit in Coleridge. After three weeks of talk he decided not to take his degree, and returned to Bristol where Coleridge joined him to share rooms in College Street. Southey introduced Coleridge to his Bristol friends, Robert Lovell and the three Fricker sisters, daughters of a failed sugar pan manufacturer of Westbury-on-Trym. The party for the Susquehanna grew but they still had to raise £125 each.

Joseph Cottle, a local bookseller, arranged some public lectures. Coleridge was asked to talk on the Rise and Decline of the Roman Empire but failed to attend. The next day he and Southey went on a visit with Cottle and their fiancées to Tintern, a quarrel arose over the missed lecture. Coleridge and Southey drifted apart as friends but shortly became brothers-in-law. Southey married Edith Fricker in St. Mary Redcliffe and went to Lisbon. Coleridge married Sarah a month later and moved to a cottage in Nether Stowey provided by his wealthy patron Thomas Poole.

Around this time Wordsworth arrived in Bristol to stay with John Pretor Pinney, the father of Montagu's pupils, a prosperous sugar merchant with plantations in Nevis in the West Indies, who lived in Great George Street. Wordsworth may have met Coleridge there or at a lecture. They corresponded. Wordsworth came to Bristol on several occasions 'to visit those extraordinary young men Southey and Coleridge'. Of Coleridge he wrote 'his talents seem very great' and Dorothy wrote that 'his conversation teems with mind, soul and spirit'. In June 1797, Coleridge visited Racedown, stayed three weeks and persuaded William and Dorothy to return with him to Nether Stowey. They soon found a house on the edge of the Quantocks to rent, Alfoxden, now a hotel.

The interaction of William and Dorothy at Racedown and of the Wordsworths and Coleridge at Nether Stowey led to a creative period. Wordsworth provided some of the ideas for 'The Ancient Mariner'. In a farmhouse between Porlock and Linton, a quarter of a mile from Culbone church, Coleridge took two grains of opium to check a dysentery. He had a visionary dream and the next morning began to write the unfinished poem 'Kubla Khan' – unfinished because of interruption by a visitor from Porlock.

'In Xanadu did Kubla Khan
A stately pleasure dome decree
Where Alph the sacred river, ran
Through caverns measureless to man
Down to a sunless sea.'

The activities of Coleridge, Wordsworth and their visiting friends gave rise to suspicion. A local doctor wrote to the Home Secretary. A secret agent was sent down and overheard them speaking about Spinoza and misinterpreted this as the Spy Nozy. He eventually concluded that they were no more than dissatisfied Englishmen. It is probably because of this that the lease on Alfoxden was not renewed in the summer of 1798.

A trip to Germany was planned and to raise money 'Lyrical Ballads' was compiled. William and Dorothy made a trip to the Wye Valley leaving Shirehampton on 9th July, crossing the Aust Ferry, staying at Tintern, walking to Goodrich, returning to Tintern and Chepstow, then back to Tintern and the next day by boat to Bristol. On 13th July as he walked down the road from the Downs into Bristol, Wordsworth completed the last twenty lines of his poem 'Tintern Abbey': 'Lines composed a few miles above Tintern Abbey on revisiting the banks of the Wye' which was unique in requiring no revision once written when he reached Bristol.

Tintern Abbey, Gwent.

'Five years have passed . . . and again I hear
These waters, rolling from their mountain-springs
With a soft inland murmur' . . .

'O sylvan Wye, thou wanderer through the woods,
How often has my spirit turned to thee.'

'. . . For I have learned
To look on nature, not as in the hour
Of thoughtless youth; but hearing oftentimes
The still, sad music of humanity,
Nor harsh nor grating, though of ample power
To chasten and subdue. And I have felt
A presence that disturbs me with the joy
Of elevated thoughts: a sense sublime
Of something far more deeply interfused,
Whose dwelling is the light of setting suns,
And the round ocean and the living air,
And the blue sky, and in the mind of man;
A motion and a spirit, that impels
All thinking things, all objects of all thought,
And rolls through all things. Therefore am I still
A lover of the meadows and the woods,
And mountains . . .'

Wordsworth as in 'Tintern Abbey' sees the supernatural in the commonplace. Coleridge as in the 'Ancient Mariner' takes the supernatural and treats it as if real.

In the winter of 1798–99 Wordsworth, shivering in lodgings in Goslar, Germany, read widely and began the poem of his life, 'The Prelude', dedicated to Coleridge and not to be published until his death in 1850. We have heard short passages from this in the description of childhood and student life. William and Dorothy returned to England early in 1799 and spent the latter part of the year with the Hutchinsons on a farm at Sockburn-on-Tees. There Coleridge joined them in October and he and William set off on a walking tour to the Lake District and it was then that Wordsworth saw Dove Cottage at Townend,

Dove Cottage, Grasmere.

Grasmere. Late in December 1799 at the stroke of the new century he and Dorothy moved in and they were to stay there for the next eight years, during which time some of the finest of English poetry was written. It was perhaps the fulfilment of a dream in an oak grove on the outskirts of Bristol when Wordsworth had a vision of a cottage in a chosen vale, its porch, its casement and its curling smoke.

Dorothy continued her journal begun at Alfoxden and this and her letters give a picture of life at Dove Cottage, the visitors and the comings and goings with Greta Hall, Keswick, where Coleridge and his family moved in 1800 to be joined by Southey and his family and the third Fricker sister, now widowed, in 1803. The shared community planned for the Susquehanna was now on the banks of the Derwent.

There were still links with Bristol through Thomas Beddoes and Humphry Davy. Beddoes was born in Shropshire in 1760, educated at Pembroke College, Oxford and received his medical education in London and Edinburgh. He was appointed Reader in Chemistry at Oxford but resigned in 1792 partly because of his unpopularity in expressing sympathy with the French Revolution and in this he was at one with Wordsworth, Coleridge and Joseph Priestley who had emigrated in 1791 after his house in Birmingham had been burnt down by an enraged mob. Professor Bruce Perry, in this Society's *Journal* January/April 1983, tells us how Beddoes came to Clifton in 1793 and of the establishment of the Pneumatic Institution for the treatment of diseases by inhalation in 1798. Wedgwood was a benefactor, Watt constructed the apparatus, Davy was engaged as an assistant.

Davy, later Sir Humphry, was born in Penzance in 1774. From boyhood he was a lover of poetry and in adolescence composed many verses and ballads. Beddoes met him when visiting Cornwall to study the geology of the coast. Davy came to Clifton in 1798. He became a friend of Coleridge and Southey and was persuaded by Coleridge to proof read the second edition of Lyrical Ballads. In 1800 Wordsworth began a correspondence asking him to correct the punctuation – 'a business of which I am ashamed to say I am not adept'. In 1804 Davy visited Dove Cottage and in 1805 climbed Helvellyn with Wordsworth and Walter Scott but became bored with the conversation and the slow progress because of Scott's lameness and made his own way back to Grasmere. Davy had by then left Bristol and had for three years been lecturer and then Professor at the Royal Institution and was to be knighted in 1812 and to become President of the Royal Society in 1820.

In 1800 Coleridge wrote a description of Wordsworth's intermittent abdominal pain to Beddoes who sent prescriptions. Coleridge wrote: 'I saw his countenance darken and all his hopes vanish when he saw the prescription – such scepticism concerning medicines!' It seems likely that the pain, sometimes associated with headache and occurring at times of stress, arose from an irritable gut. No more serious disease seems to have developed and we are told that his fancied ills were all forgotten in the overwhelming sorrow of his daughter Dora's death in July 1847.

Dorothy Wordsworth's friend Catherine Clarkson of Ullswater had travelled to Bristol to consult Dr. Beddoes. Dorothy wrote: 'Surely Dr. Beddoes when he sees you and hears from yourself the history of your symptoms, your uprisings and your fallings, will be able to ascertain the course of your disease.' Southey wrote of Clarkson 'he is piping hot from Bristol and brimful of admiration for Beddoes who indeed seems to have done so much for Mrs. Clarkson that there are hopes for her speedy recovery.' Encouraged by this, Dorothy asked Mrs. Clarkson to tell Dr. Beddoes of her own symptoms and to ask what might be done in an attack. 'I begin with sickness, violent headaches, yellow and pale looks and afterwards pain in the bowels, thirst and want of appetite'. A prescription was sent and dispensed in Keswick, and Dorothy writes that she will attend to advice respecting diet and take medicine regularly. 'What a good man and what a good physician Dr. Beddoes is. I shall reverence him as long as I live.' It is probable that her illness was gallstones with possible diverticulitis. Despite Dr. Beddoes the attacks continued and there was particularly troublesome internal inflammation in 1829 in which she lay for two days in excruciating torture. There were further relapses and then mental and physical decline over 20 years following influenza in 1835.

There was an unusually close relationship between William and Dorothy. He says:

'She gave me eyes, she gave me ears
And humble care and delicate fears,

A heart, the fountain of sweet tears
And love and thought and joy.'

Dorothy describes a walk on the shores of Ullswater in 1802. 'I never saw daffodils so beautiful, they grew among the mossy stones and about them, some rested their heads among these stones as on a pillow and the rest tossed and danced and reeled.' William in 1804 writes:

'I wandered lonely as a cloud
That floats on high o'er vales and hills,
When all at once I saw a crowd,
A host, of golden daffodils;
Beside the lake, beneath the trees,
Fluttering and dancing in the breeze.

For oft, when on my couch I lie
In vacant or in pensive mood,
They flash upon that inward eye
Which is the bliss of solitude;
And then my heart with pleasure fills,
And dances with the daffodils.'

Dorothy describes a walk up Greenhead Ghyll looking for a sheep fold. Wordsworth based on this the poem 'Michael.'

If from the public way you turn your steps
Up the tumultuous brook of Green-head Ghyll,
You will suppose that with an upright path
Your feel must struggle; in such bold ascent
The pastoral mountains front you, face to face.
But courage! for around that boisterous brook
The mountains have all opened out themselves,
And made a hidden valley of their own.'

Davy considered this poem about Michael, the shepherd, at 84 still strong and hale, out each day for the next seven years with his faithful dog building a yet unfinished sheep fold to be full of just pictures of what life ought to be.

In July 1802, four months after the Peace of Amiens, William and Dorothy travelled to France to meet Annette at Calais. They passed through London. Dorothy wrote: 'It was a beautiful morning. The City, St. Paul's with the river and a multitude of little boats made a most beautiful sight as we crossed over Westminster Bridge.' William wrote:

'Earth has not anything to show more fair:
Dull would he be of soul who could pass by
A sight so touching in its majesty:
This City now doth, like a garment, wear
The beauty of the morning; silent, bare,
Ships, towers, domes, theatres, and temples lie
Open unto the fields, and to the sky;
All bright and glittering in the smokeless air.
Never did sun more beautifully steep
In his first splendour, valley, rock, or hill;
Ne'er saw I, never felt, a calm so deep!
The river glideth at his own sweet will:
Dear God! the very houses seem asleep;
And all that mighty heart is lying still!'

Shortly after returning from France William married Mary Hutchinson. They had met as children in Penrith. Mary had visited Racedown. William had stayed with Mary and her family at Sockburn-on-Tees on his return from Germany and she had visited Dove Cottage. Dorothy wrote to a friend 'I have long loved Mary as a sister, but happy as I am I half dread that concentration of all tender feelings past, present and future which will come upon me on the wedding morning'. In the event she could not face going to the church. After the wedding the three of them returned to Dove Cottage, soon to be joined by Mary's sister Sarah.

William wrote of Mary:

'She was a Phantom of delight
When first she gleamed upon my sight:
A perfect Woman, nobly planned
To warn, to comfort and command
And yet a Spirit still and bright
With something of angelic light.'

Their son John was born in 1803, Dora 1804, Thomas 1806, Catherine 1808, and soon Dove Cottage was too small for the family and their many visitors and in 1808 they moved to Allan Bank, where their youngest child, Willy, was born in 1810. In 1811 they moved to the Parsonage. There Catherine died from convulsions in June 1812. She had been lame following a convulsion a year earlier and it seems likely that this was some vascular incident, and not poliomyelitis as had been suggested by some biographers. In December of that year, Thomas died from pneumonia complicating measles. They were now desperate to move from the Parsonage with its unhappy associations and on 1st May 1813 took the tenancy of Rydal Mount where they were to be for the rest of their lives. Wordsworth took his first paid appointment as Distributor of Stamps for Westmorland – and settled into a contented family life, a landscape designer, gardener, traveller and travel writer. Though he wrote much he only occasionally recaptured the vision of his creative period, and sensed this when he wrote 'Intimations of Immortality':

'There was a time when meadow, grove, and stream,
The earth, and every common sight,
To me did seem
Apparelled in celestial light,
The glory and the freshness of a dream.
It is not now as it hath been of yore: –
Turn wheresoe'er I may,
By night or day,
The things which I have seen I now can see no more.'

The present garden at Rydal Mount is much the same as that which Wordsworth made, and in the last five years has been carefully restored by Mr. and Mrs. Don Brooks, curators of Rydal Mount. When William moved in there was a Mount, said to be a Norse lookout with views to Loughrigg and Wansfell:

Summerhouse, Rydal Mount.

Rydal Water from Rydal Mount.

'Wansfell, this household has a favoured lot
with liberty on thee to gaze.'

Wordsworth built a terrace leading from the house to a summer house where he used to sit murmuring verses or looking out over Rydal Water. The Westmorland Directory of 1829 refers to Rydal Mount 'where Wordsworth spent the greater part of his life in scenery which in grandeur and beauty is scarcely equalled in Westmorland and Cumberland. The house and gardens are in the best taste, the latter having assumed their present form under the poet's own hand.' There is a fine description of the garden in *Country Life,* 3rd May 1984, pp. 1240–1242.

Wordsworth's fame grew. He received honorary degrees at Durham and Oxford in 1838 and 1839, had many visitors among them the Dowager Queen Adelaide in 1840, and in 1843 succeeded Southey as Poet Laureate. Peel wrote: 'I understand that you will have nothing required of you. As the Queen can select for this honourable appointment no-one whose claims for respect and honour on account of eminence as a poet can be placed in comparison with yours. I trust you will no longer hesitate to accept.' In 1845 he was presented to Queen Victoria at a Fancy Dress Ball which he attended in full court dress.

He felt the sadness of bereavement. Walter Scott 1832, Coleridge 1834, Charles Lamb 1834, James Hogg the Ettrick Shepherd poet in 1835 – and his death led to the best of his later verse, composed in half-an-hour and written, like Tintern Abbey 37 years earlier, without alteration:

'The mighty Minstrel breathes no longer,
'Mid mouldering ruins low he lies;
And death upon the brass of Yarrow,
Has closed the Shepherd-poet's eyes:

Nor has the rolling year twice measured,
From sign to sign, its steadfast course,
Since every mortal power of Coleridge
Was frozen at its marvellous source;

The rapt One, of the godlike forehead,
The heaven-eyed creature sleeps in earth:
And Lamb, the frolic and the gentle,
Has vanished from his lonely hearth.

Yet I, whose lids from infant slumber
Were earlier raised, remain to hear
A timid voice, that asks in whispers.
"Who next will drop and disappear?"'

Wordsworth, like the Shepherd Michael, was active in old age. He made a trip to Italy at the age of 67. He went on long walks and at 75 was helping with the hay-making. He realised as an old man that the character of youth and age were similar. The inherent variety and originality of youth merged into dull uniformity in middle age but then reappeared afresh in old age. Like trees, the real characteristics only appear in spring and autumn.

His last years were saddened by the physical and mental decline of his sister Dorothy following influenza in 1835. She never regained her mind and lived in a twilight world confined to a wheelchair able only to walk a few steps. She was determined to sit roasting in front of a fire even on the hottest day. There were frequent outbursts of anger with shouts and screaming, but also lucid spells. She was usually able to complete quotations. Her memory for past events was unimpaired and at times she could express opinions with good judgement. At the time of William's death in 1850 she was quiet, self-possessed, full of consideration for his wife and as she passed his door in her wheelchair, she murmured: 'Oh Death, where is thy sting? Oh Grave, where is thy victory?' – but relapsed again into her usual querulous condition. She died in January 1855.

Dorothy Wordsworth. Portrait by S. Crosthwaite, 1833.

What was her condition? It is usually attributed to arteriosclerosis, but could it be hypothyroidism, Altzheimer's disease or a complication of influenza? I have taken the advice of Professor Gordon Wilcock, Professor in Care of the Elderly and I am grateful for his comment, on what must be quite inadequate evidence. The possibilities are a meningo-encephalitis following influenza with brain damage, or normal pressure hydrocephalus, for difficulty in walking is a late feature of other dementias. Hypothyroidism would be an attractive possibility but she should have lapsed into myxoedema coma and perished well before the 20 years were up. Perhaps it was a psychiatric condition or even acute porphyria.

There was even deeper sadness in the death of William's daughter Dora in July 1847 probably from tuberculosis of the spine.

In 1849 Wordsworth visited Malvern, and travelled back by train and along the new railway from Oxenholm to Windermere whose building he had opposed in 1844:

> 'Is then no nook of English ground secure
> From rash assault?'

In March 1850 he took a short walk round Grasmere, developed pleurisy, and his condition worsened. He died on Tuesday, 23rd April 1850 – Shakespeare's and England's Day – at 12 noon exactly as the cuckoo clock was striking the hour.

Russell Brain in his essay 'Some Reflections on Genius' writes: "The poet uses words to evoke images and images to move and delight, new combinations of words to shock his reader into new experience, or to revivify old ones, to excite pleasure by virtue of rhythm, rhyme and assonance.' He considers that the inspiration of the poet is of the same nature as that which leads to scientific discovery, differing only in its subject matter. It may, we might think, have similarity to good medical diagnosis.

"The precise and intelligent recognition and appreciation of differences is the real essential factor in all successful medical diagnosis. Eyes and ears which can see and hear, memory to recall at once and to recall with pleasure the impression of the senses and an imagination capable of weaving a theory or piecing together a broken chain or unravelling a tangled clue, such are the implements of his trade to a successful diagnostician.'

Joseph Bell 1839–1911.

Wordsworth considers poetry to be the spontaneous overflow of powerful feelings. It takes its origin in 'emotion recollected in tranquillity'. Emotion is regenerated and in this mood composition begins. He speaks of inspiration as a gentle breeze blowing from the green fields and the clouds and from the sky, exciting an inward creative breeze which grows and becomes like a tempest.

The reaction to poetry may be equally powerful. A. E. Housman had learned from experiences to keep watch over his thoughts when shaving, for if a line of poetry strayed into his memory, his skin bristled so that the razor ceased to act.

Philip Larkin writes that Wordsworth was nearly the price of him. Driving down the M1 in the middle lane at 70 miles an hour, someone on the radio read the Immortality Ode '. . . There was a time when meadow, grove and stream . . .' and he could not see for tears.

Wordsworth in a letter of 1807 said of his poems that they were to console the afflicted, to add sunshine to daylight by making the happy happier, to teach the young of every age to see, to think and feel.

We see in this story much of importance to the study of old age. The old men of Hawkshead, the Shepherd Michael, the deaths in childhood of Catherine and Thomas, and in middle life of Wordsworth's parents and Dora, reminding us that there are more old people now not because people live longer but because fewer people die in childhood and adult life. We see the twilight state in which Dorothy Wordsworth lived and wonder why this must be, and how this might have been prevented in her and in our own patients. We sense the sadness of bereavement in the Extempore Effusion on the Death of James Hogg. We see Wordsworth healthy and active in old age considering that old age had much of the vitality of youth, and able still to appreciate the beauty of the rainbow:

'My heart leaps up when I behold
A rainbow in the sky:
So was it when my life began:
So is it now that I am a man;
So be it when I shall grow old,
Or let me die!
The Child is Father of the Man;
And I could wish my days to be
Bound each to each by natural piety.'

### BIBLIOGRAPHY

BRAIN, W. R. (1961) Some Reflections on Genius and other Essays. Pitman.

DAVIES, H. (1980) A Walk around the Lakes. Hamlyn Paperbacks.

DAVIES, H. (1981) William Wordsworth. Hamlyn Paperbacks.

DE QUINCEY, T. (1980) Recollections of the Lakes and the Lake Poets. Penguin Books.

HOWE, H. W. (1977) Greta Hall, Home of Coleridge and Southey. Daedalus Press.

McCRACKEN, D. (1984) Wordsworth and the Lake District. A Guide to the Poems and their Places. Oxford University Press.

MOORMAN, M. (1957, 1965) William Wordsworth. Vol. 1 Early Years, Vol. 2 Later Years. Oxford University Press.

RAWNSLEY, H. D. (1901) Literary Associations of the English Lakes. James MacLehose & Sons.

READ, H. (1965) Wordsworth. Faber and Faber.

Wordsworth, Dorothy, Journal of (1971). Moorman, M. (ed.). Oxford University Press.

Wordsworth, Dorothy, Letters of (1981). Hill, A. G. (ed.). Oxford University Press.

WORDSWORTH, WILLIAM (1981) Poetical Works. Oxford University Press.

WORDSWORTH, WILLIAM (1969) Poetry and Prose. Nichol Smith, D. (ed.). Oxford University Press.

WORDSWORTH, WILLIAM (1981) Selected Poems. Sharroch, R. (ed.). Heinemann.

WORDSWORTH, WILLIAM (1982) Guide to the Lakes (1835). De Sélincourt Ernest (ed.). Oxford University Press.

WORDSWORTH and COLERIDGE (1983) Lyrical Ballads (1798) Owen, W. J. B. (ed).) Oxford University Press.

WORDSWORTH and COLERIDGE (1982) Lyrical Ballads (1805). Roper, D. (ed.). MacDonald and Evans.

Wordsworth, William, Letters of (1984). Hill, A. G. (ed,). Oxford University Press.

Wordsworth, William and Mary, Love Letters of (1982). Darlington, B. (ed.). Chatto and Windus.

## Figures and Tables

**Figure f1:**
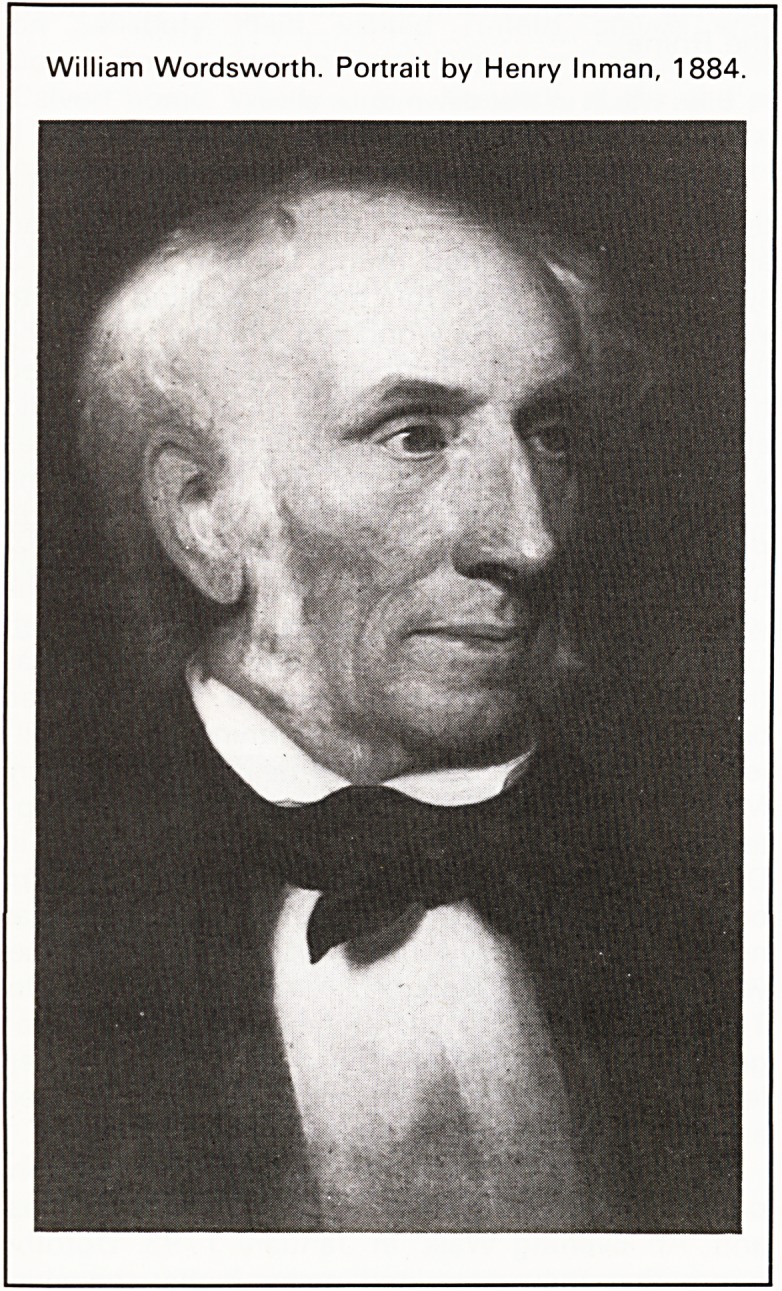


**Figure f2:**
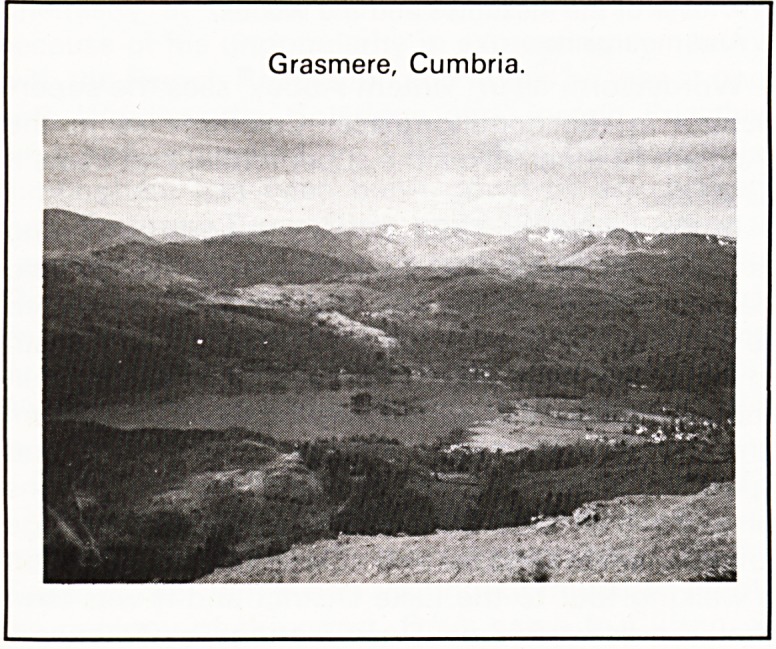


**Figure f3:**
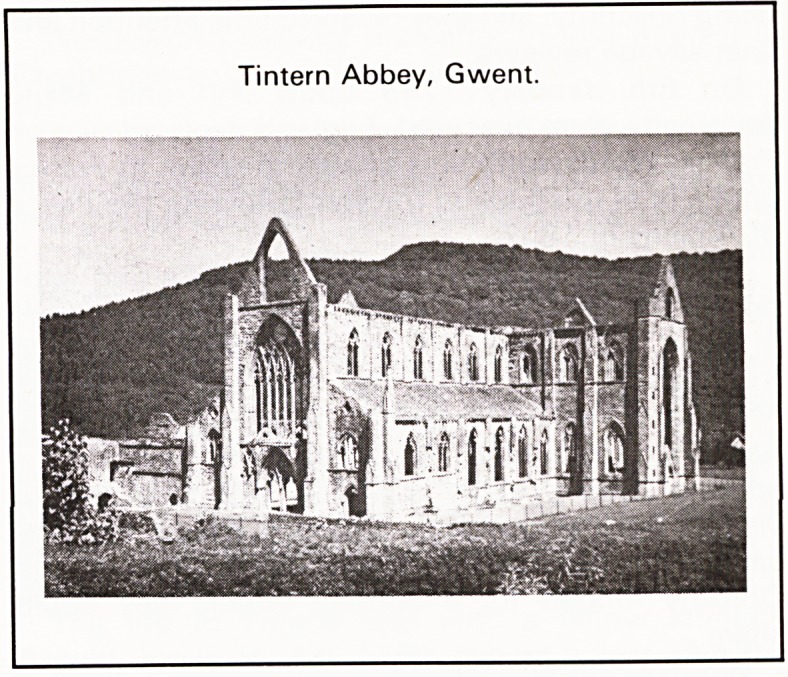


**Figure f4:**
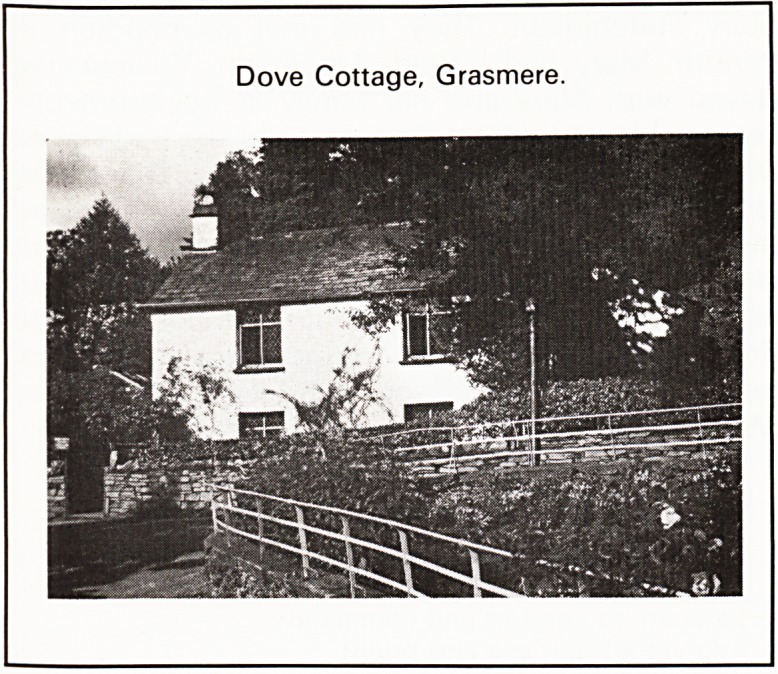


**Figure f5:**
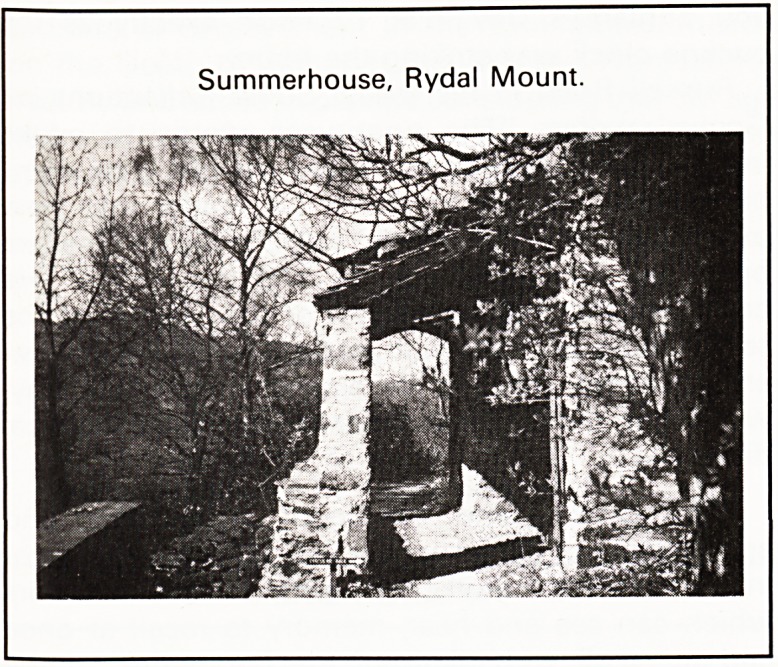


**Figure f6:**
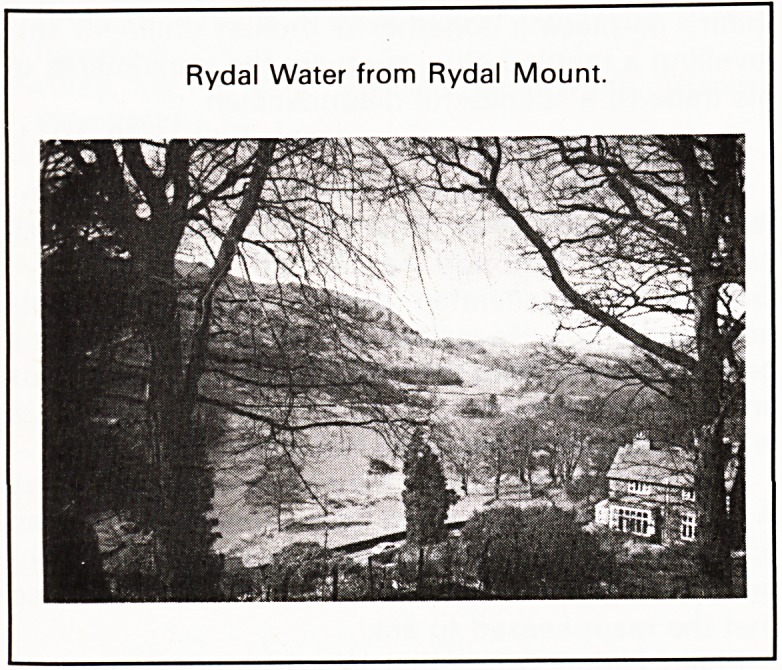


**Figure f7:**